# Extraction of Perchlorate Using Porous Organosilicate Materials 

**DOI:** 10.3390/ma6041403

**Published:** 2013-04-02

**Authors:** Brandy J. Johnson, Iwona A. Leska, Brian J. Melde, Ronald L. Siefert, Anthony P. Malanoski, Martin H. Moore, Jenna R. Taft, Jeffrey R. Deschamps

**Affiliations:** 1 Center for Bio/Molecular Science and Engineering, Naval Research Laboratory, Washington, DC 20375, USA; E-Mails: brian.melde@nrl.navy.mil (B.J.M.); anthony.malanoski@nrl.navy.mil (A.P.M.); martin.moore@nrl.navy.mil (M.H.M.); jeffrey.deschamps@nrl.navy.mil (J.R.D.); 2 NOVA Research Incorporated, Alexandria, VA 22308, USA; E-Mails: iwona.leska.ctr@nrl.navy.mil (I.A.L.); jennartaft@gmail.com (J.R.T.); 3 Chemistry Department, US Naval Academy, Annapolis, MD 21402, USA; E-Mail: siefert@usna.edu

**Keywords:** periodic mesoporous organosilica, perchlorate, solid phase extraction, groundwater monitoring

## Abstract

Sorbent materials were developed utilizing two morphological structures, comprising either hexagonally packed pores (HX) or a disordered pore arrangement (CF). The sorbents were functionalized with combinations of two types of alkylammonium groups. When capture of perchlorate by the sorbents was compared, widely varying performance was noted as a result of differing morphology and/or functional group loading. A material providing improved selectivity for perchlorate over perrhenate was synthesized with a CF material using *N*-trimethoxysilylpropyl-*N*,*N*,*N*-trimethylammonium chloride. Materials were applied in batch and column formats. Binding isotherms followed the behavior expected for a system in which univalent ligands of varying affinity compete for immobilized sites. Performance of the sorbents was also compared to that of commercial Purolite materials.

## 1. Introduction

Compounds such as nitroenergetics and perchlorates, used as components of common ordnance, present concerns regarding contamination of water sources at sites associated with US Department of Defense (DoD) activities. Perchlorate is soluble in water and is repelled by the negative charge of soil particles resulting in high mobility. As a result, perchlorate contamination presents a potential health hazard to military personnel and their families residing on DoD installations as well as to surrounding populations and wildlife [1,2]. Perchlorate contamination is not, however, isolated to DoD sites or solely a result of DoD activities. Rising levels in drinking water are a current concern for the US Environmental Protection Agency (EPA) [3]. Perchlorate is a naturally occurring compound found as a result of atmospheric deposition in arid environments [4]. It has also been mined and transported as a natural contaminant in nitrate fertilizer, and it is manufactured for use as a propellant, in fireworks, and in medicine [5]. Because of the wide range of potential contributing factors, disputes regarding contamination sources are inevitable. It has been demonstrated that sources of perchlorate contamination can be discriminated on the basis of isotopic ratios [6,7]. Analysis typically involves extraction of perchlorate using an anion-exchange resin [8].

We have previously described our efforts focused on the development of organosilicate materials for preconcentration of nitroenergetic compounds [9,10,11,12]. Sorbents have also been developed for the capture of organophosphate and solvent targets [13,14,15]. While binding of nitroenergetic targets can be accomplished through interactions with the sorbent surfaces (hydrophobic/hydrophilic interactions, π–π stacking, *etc*.), capture of perchlorate targets requires a different approach. Alkylammonium groups have been described previously for this purpose [16], and there are commercially available resins based on the idea (Purolite, Bala Cynwyd, PA). Unfortunately, ion exchange is, in general, nonspecific resulting in the capture of, not only perchlorate, but competing ions in the sample. These sorbents are, as a result, less than ideal for solid phase extraction of perchlorate and must be combined with additional processing steps for removal of the resulting concentrated competing ions. In addition, commercial sorbents utilize an acidic iron solution (FeCl_4_^−^) for target elution. These types of solutions require further processing steps, including Fe_3_^+^ precipitation, prior to analysis by ion chromatography (IC) [7]. Cationic components may also impact suppressor performance in IC systems [17]. 

Here, a series of porous silicate materials designed based on a previously described synthetic approach [18] was evaluated for perchlorate capture. The focus of the effort was development of a sorbent applicable to preconcentration of perchlorate that provided high affinity for perchlorate as well as preferential binding of perchlorate over compounds typically found as co-contaminants. High total binding capacity for perchlorate was desirable, but could be sacrificed to some extent in favor of selectivity. It was also of interest to develop a sorbent that would allow for elution of the target under less restrictive conditions than those offered by commercially available materials. Varied levels of *N*-trimethoxysilylpropyl-*N*,*N*,*N*-trimethylammonium chloride (TSPMC) and *N*-trimethoxysilylpropyl-*N*,*N*,*N*-tri-n-butylammonium chloride (TSPBC) were loaded into organosilicate scaffolds of differing morphology through post-synthesis grafting. Target binding by and elution from these sorbents is characterized. Comparisons to commercially available sorbents, Purolite A530E and A532E, are provided.

## 2. Results and Discussion

**Figure 1:**
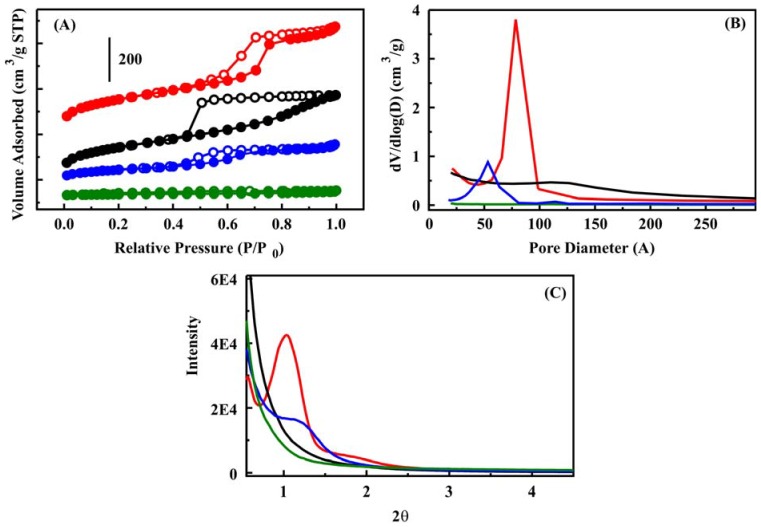
(**A**) Nitrogen sorption isotherms for sorbent with hexagonally packed pores (HX) (red, shifted +200), sorbent with disordered pore arrangement (CF) (black, shifted +110), HX1M3B (blue), and CF1M3B (green) sorbents; (**B**) Pore size distributions for HX, CF, HX1M3B, and CF1M3B. The inset shows a zoom view of the HX1M3B and CF1M3B pore size distributions; (**C**) X-ray diffraction patterns for HX (red), CF (black), CF2 (blue), and CF3 (green) sorbents.

**Figure 2:**
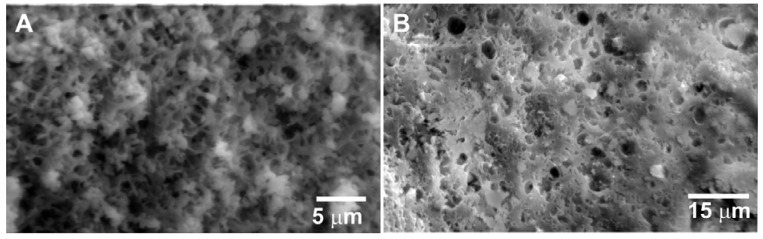
Scanning electron microscopy (SEM) images of HX (**A**) and CF (**B**) showing differing macroscale morphologies.

Materials were designed with the intention of their use in a column format for capture of analyte from a flowing solution. Ideally, such a material should combine high surface area and dense population of binding sites with a texture that enables facile diffusion of solvent through the length of column. The syntheses used here combined surfactant templating of mesostructured materials and polymerization-induced phase separation to create macroscale textures [19]. An alternative approach might include the use of materials with very large mesopores to improve diffusion through the sorbent materials [20,21]. The protocols for synthesis of HX and CF were adapted from the literature to provide macroporous silica supports offering significantly different mesostructures [18,19]. HX was synthesized with the intention of obtaining hexagonal packing of cylindrical pore channels. CF was intended to be a “mesostructured cellular foam” with a relatively disordered arrangement of spherical pores. These morphological considerations are evident in their nitrogen sorption isotherms (Figure 1). HX showed a steep increase in adsorption around *P*/*P*_0_ = 0.7 and a parallel hysteresis between the adsorption and desorption branches. This is in contrast to the pronounced hysteresis observed for CF and the increase in adsorption extending over a wide relative pressure region (*P*/*P*_0_ = 0.5–1.0). HX yielded a narrow pore size distribution peak at 77 Å (Figure 1). The pore size distribution of CF was less defined and peaked ca. 111 Å. CF did not provide reflections in a powder X-ray diffraction pattern (Figure 1). HX demonstrated a more crystalline pattern having a primary reflection at 2*θ* = 1.0° and a secondary reflection *ca*. 2*θ* = 1.8°. Open, co-continuous macropores in a strut-like framework were observed for the HX material (Figure 2). CF, however, displayed an inconsistent morphology comprised largely of bulk particles with some regions of open macroporosity (Figure 2). 

Post-synthesis grafting of quaternary alkylammonium groups onto these sorbents was used to provide sites for perchlorate anion binding. The naming scheme used the scaffold abbreviation (HX or CF) followed by the quantity of functional compound utilized in the grafting step (mmol), so 1 g of CF functionalized with 4 mmol of TSPMC chloride was abbreviated CF4M while functionalization with 4 mmol of TSPBC would be abbreviated as CF4B. The selection of the alkylammonium groups was based on the reported performance of an ordered mesoporous SBA-15 silica grafted with TSPMC and TSPBC [16]. SBA-15 typically consists of rope-like particles of about 1 mm in size [22]. The sorbents used here, HX and CF, offered both meso and macrostructures that differentiate them from the previously evaluated SBA-15 (Figure 1 and Figure 2). Grafting of the TSPMC and TSPBC functional groups, results in changes in the nitrogen sorption characteristics of the sorbents (Table 1). Reacting 1 g of HX material with up to 4 mmol of ammonium silanes did not compromise its mesoporosity (Figure 1). Pore sizes > 60 Å and surface areas exceeding 100 m^2^/g were measured. Loading of CF with such high amounts of ammonium silanes, however, resulted in low surface areas around 25 m^2^/g (Figure 1). Previously evaluated SBA-15 materials utilized 4 mmol per gram loading of the functional groups through incipient wetness impregnation and reported losses in surface area and pore volume of 50% to 75%. Quantitative loading was assumed for the sorbents in that study [16]. 

### 2.1. Batch Binding Experiments

Generation of CF and HX sorbents with various combinations of surface functional groups initially provided twelve materials. In order to select those most likely to provide desirable binding characteristics, batch binding experiments using sorbent masses of 15 and 30 mg (5 mL, 500 ppm solution) were performed for perchlorate (Table 1). Based on the previous report [16], it was expected that combinations of the two functional groups would provide increased target binding. It was also expected that higher loading levels would be advantageous at least to the point at which transport of the targets through the sorbents was negatively impacted. Of the twelve sorbents initially evaluated, HX2M bound the most perchlorate from the batch experiment. Neither loading of the sorbent using 4 mmol of the TSPMC nor addition of 2 mmol TSPBC increased the total target bound. Both of these modifications decreased the surface area of the sorbent (27% and 48%, respectively) and likely restricted access to significant portions of the pore volume. When perchlorate binding by HX4M and HX4B was compared to that by HX1M3B and HX2M2B, increased binding was observed for the mixed functional groups. This point was interesting considering the surface area of the mixed functional group sorbents is 27% less than that of the HX4M sorbent. It is important to note that the surface area, as determined by nitrogen adsorption, may not reflect that accessible by the ionic targets under consideration here. Mobility of surface functional groups and permeability through the organosilane layer may be different under aqueous conditions than under the conditions used for morphological analysis. 

In the per gram comparison, HX sorbents largely outperformed the CF variants. HX sorbents with 2 mmol functional group bound perchlorate at levels similar to those of the 4 mmol CF variants. On closer inspection, it was interesting to note that the surface area of the 4 mmol CF variant was an order of magnitude less than that of the 4 mmol HX variant. The HX1M3B and CF1M3B sorbents bound equivalent amounts of perchlorate per gram while their respective surface areas would tend to indicate much greater site availability on the HX sorbent. When target bound was normalized to surface area, the results had a dramatically different appearance (Table 1). CF4M and CF1M3B bound nearly ten times more perchlorate per unit surface area than the other sorbents initially evaluated. There are several possible causes for these differences. It is possible that the HX sorbents have a lower degree of functionality, *i.e.*, a smaller fraction of the functional groups are bound during grafting of the sorbent. It is also possible that sites within the HX sorbent are subject to different restrictions and steric hindrance resulting in a lower activity and/or that regions of the HX sorbent that are penetrated by nitrogen (used for calculation of surface area) are not accessible to the ionic target. Determining the specific factors influencing the results was not possible with the available analyses.

Due to the possible differences between determined surface area and that accessible to the ionic targets, surface area normalization of the data was not appropriate to selection of a final candidate. This type of normalization, however, can provide insights useful in additional materials development. On the basis of those results, the CF sorbent was redesigned through modification of the synthetic protocol to provide two new variants CF2-4M and CF3-4M (Table 1) with increased nitrogen accessible surface area. CF2 had a narrow pore size distribution similar to the HX materials peaking at 48 Å. The CF3 sorbent had the wide H2-type hysteresis loop observed for CF. The CF3-4M sorbent bound 13% more perchlorate than the CF4M sorbent with only a slight increase in surface area. It is possible that the total target binding capacity could be further increased through improved accessibility to the pore volume. This was not, however, pursued as it is not the only consideration for this effort. In addition to providing binding capacity for perchlorate, this effort sought to provide preferential binding for perchlorate over other environmental targets such as perrhenate. With that in mind, similar batch binding experiments were conducted for nitrate, perrhenate, thiocyanate, sulfate, and phosphate using 10 mg of sorbent in 200 ppm target solutions (5 mL). The results are provided in the Supporting Information (Tables S1 and S2). 

**Table 1:** Morphological characteristics and target binding for base and alkylammonium functionalized sorbents with a disordered pore arrangement (CF) and with hexagonally packed pores (HX).

Material	Surface Area (m^2^/g)	Pore Volume (cm^3^/g)	Pore Diameter (Å)	[Functional Group] (mmol) ^%^	Perchlorate Bound (mg) *	Perchlorate Bound (μg/m^2^)
**HX Products**						
HX	566	0.70	77	–	–	–
HX05M05B	342	0.43	64	1	–	–
HX2M	321	0.47	64	2	1.80	187
HX1M1B	206	0.29	63	2	1.25	202
HX4M	232	0.36	64	4	1.35	194
HX4B	342	0.34	63	4	1.33	130
HX2M2B	169	0.26	63	4	1.42	280
HX1M3B	163	0.22	53	4	1.66	339
HX2M4B	226	0.34	60	6	0.37	55
**CF Products**						
CF	523	0.57	111	–	–	–
CF05M05B	236	0.36	111	1	–	–
CF2M	197	0.29	93	2	1.30	220
CF1M1B	239	0.30	93	2	1.11	155
CF4M	25	0.04	111	4	1.73	2,310
CF4B	185	0.38	111	4	1.29	232
CF2M2B	143	0.18	93	4	1.51	352
CF1M3B	23	0.03	111	4	1.66	2,406
CF2	520	0.56	48	–	–	–
CF2-4M	108	0.12	38	6	1.27	392
CF3	562	0.52	64	–	–	–
CF3-4M	70	0.12	64	7	1.95	929

^%^ Total functional groups (TSPMC + TSPBC) applied per gram of sorbent during grafting step; * By 30 mg of sorbent from 5 mL of 500 ppm solution.

Overall, binding ratios varied widely for different ions on different sorbents (Supporting Information, Table S2). In these materials, each of the binding sites consists of a single alkylammonium group. Though variations in selectivity between TSPMC and TSPBC might be expected, variations in binding by TSPMC in different structures would not be. The top binding HX sorbents (HX2M and HX1M3B) demonstrated greater binding of perrhenate over perchlorate, though more perchlorate than thiocyanate was bound. Similarly, the CF sorbents bound more perrhenate than perchlorate from identical conditions. The CF2-4M and CF3-4M sorbents, however, bound at least as much perchlorate as perrhenate and bound more perchlorate than most other ions. This data tended to indicate preference for perchlorate binding by functional groups for which perrhenate preference would be expected. When the Purolite resins (A530E and A532E) were evaluated for comparison (Supporting Information, Tables S1 and S2), the resins bound significantly more perrhenate than perchlorate. It should be noted that perchlorate binding from batch experiments by these resins was several times greater than that of the organosilicate sorbents. While for larger targets, size exclusion can contribute to the binding properties of mesoporous sorbents, the large pore sizes (53 to 111 Å) and small targets (Supporting Information, Table S3) considered here make this effect unlikely. Further, the total target bound from the batch experiments is only a few percent of the total TSPMC/TPSBC used during the grafting steps, and the volume of the target bound is less than 10% of the pore volume available even in the CF4M sorbent. 

**Figure 3:**
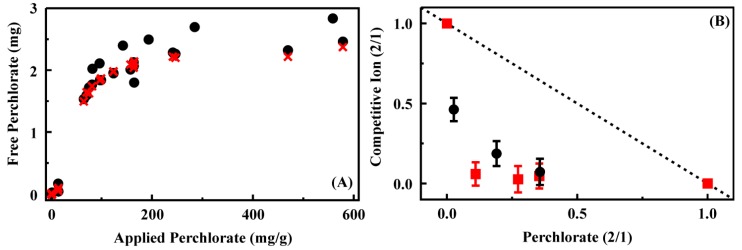
(**A**) Perchlorate binding by CF4M from batch experiments. Shown are experimental data (black circle) as well as the results of fitting that data (red ×); (**B**) Competitive ion binding from mixed target solutions by CF3-4M. Data for each axis is plotted as the ratio of the target bound from the two target solution to that bound from the single target solution: binding from solutions of perchlorate and perrhenate (black circles) and binding from solutions of perchlorate and thiocyanate (red squares). Perchlorate binding and competitive binding results for additional sorbents provided in the Supporting Information, Figures S4 and S5.

Batch experiments were completed to provide more thorough characterization of selected materials. Binding isotherms were generated based on a system in which univalent ligands of varying affinity compete for immobilized sites [23]. The expression accounts for the concentration of sites and both ligands (perchlorate and chloride) as well as the affinity of each ligand for the sites. It is:

(1) $${\left[Perchlorate\right]}_{\text{Bound}}=\frac{\frac{K_{\text{P}}}{1+K_{\text{C}}{\left[Chloride\right]}_{\text{Free}}}\;{\left[Sites\right]}_{\text{Total}}\;{\left[Perchlorate\right]}_{\text{Free}}}{1+\frac{K_{\text{P}}}{1+K_{\text{C}}{\left[Chloride\right]}_{\text{Free}}}{\left[Perchlorate\right]}_{\text{Free}}}$$

where *K*_P_ and *K*_C_ are the site affinity for perchlorate and chloride, respectively. Concentrations for bound and free perchlorate and free chloride were obtained from IC data. In our implementation, site concentration was a variable to be determined where the volume and mass of sorbent were known quantities. This allowed for calculation of the sites (mole) per gram of sorbent. Figure 3 presents both the experimental data and the calculated data (perchlorate bound) based on fitting of this expression for CF4M. Additional data for other materials is provided in the Supporting Information (Figure S4) and a summary of the association constants and site loading levels is provided in Table 2. 

**Table 2:** Parameters from fits generated for perchlorate binding isotherms. Here, *K*_P_ and *K*_C_ are the site affinity for perchlorate and chloride, respectively. *X* is the effective site concentration per gram of sorbent.

Material	*K*_P_ (M^−1^)	*K*_C_ (M^−1^)	*X* (mmol/g)
**HX Products**			
HX2M	662	494	1.86
HX1M1B	1428	985	1.92
HX4M	6380	881	0.45
HX2M2B	625	844	0.91
HX1M3B	1076	112	0.79
**CF Products**			
CF2M	1424	789	1.79
CF1M1B	2738	4359	1.85
CF4M	7550	741	0.28
CF4B	8984	265	0.56
CF2M2B	776	3102	1.46
CF1M3B	833	192	1.09
CF2-4M	2348	1104	0.81
CF3-4M	6719	3974	1.74

From Table 2, the variation in affinity for a specific ligand immobilized in each of the sorbents becomes more apparent. It is also important to note that increasing the functional group loading levels does not necessarily result in additional sites in the sorbents. If site concentrations for HX2M and HX4M are compared in Table 2, HX2M has 1.86 mmol/g and HX4M has 0.45 mmol/g. This result would tend to indicate that loading of HX with 4 mmol of the alkylammonium group resulted in blockage of pores and inaccessible regions in the sorbent. The result is supported by the differences in surface area for the two materials (321 m^2^/g for HX2M *versus* 232 m^2^/g for HX4M). Another example is CF4M where site concentration is only 0.28 mmol/g and surface area has been reduced by 95% from the base material. 

Additional batch binding studies from mixtures of compounds were completed for selected materials to obtain a better understanding of the relative binding of the different ionic targets. In this adapted approach [10,24,25], total target concentration is held constant while the ratios of the ionic compounds are varied. These types of studies tend to indicate whether non-target compounds will interfere with binding of targets. They can also provide an indication of whether two compounds bind to similar sites within a sorbent. This use of the analysis is traditional when the system under consideration involves monolayer binding on surfaces. While the sorbents considered here do not fit that model (based on isotherm analysis), the approach still provides insight into competitive binding by the sorbents. As shown in Figure 3, significant amounts of thiocyanate were not bound from mixtures including perchlorate at any of the concentration ratios (4:1, 1:1, 1:4) by the CF3-4M sorbent. Perrhenate was bound from mixtures, but that binding had only a small effect on the perchlorate bound. Binding from mixed targets was highly variable across the other sorbents. CF3-4M, CF2-4M, and HX2M preferentially bound perchlorate over perrhenate or thiocyanate (additional data provided in the Supporting Information, Figure S5). 

CF4M, CF1M3B, and HX1M3B preferentially bound perrhenate over perchlorate, but bound similar amounts of perchlorate and thiocyanate. These differences capture the effects of varying the functionality of the surfaces through differing ratios of TSPMC and TSPBC. In light of the differences in results for CF4M, CF2-4M, and CF3-4M, however, those effects alone do not account for all of the factors influencing the performance of the materials. It seems likely that factors such as surface curvature and crowding at the alkylammonium group sites influence the performance of the sorbents as well.

### 2.2. Column Binding Experiments

**Figure 4:**
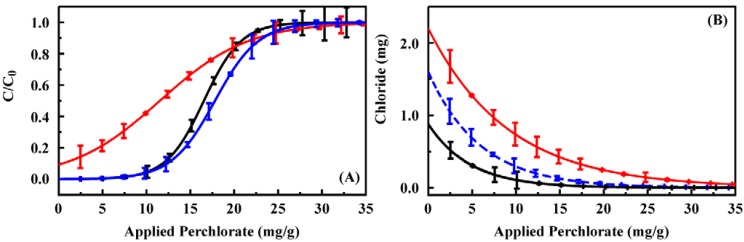
(**A**) Perchlorate breakthrough for 200 mg columns of CF4M (solid), CF2-4M (red), and CF3-4M (blue) using a 10 ppm solution at a flow rate of 1 mL/min; (**B**) Chloride recovered in volumes collected during breakthrough experiments. Similar data for a column of Purolite A530E is provided in the Supporting Information, Figure S6.

The intention is for these sorbents to be applied in column format for the capture of targets from natural water sources. Column breakthrough experiments were used to evaluate the potential of CF4M, CF2-4M, and CF3-4M (Figure 4) for this application (200 mg sorbent columns). Initial perchlorate breakthrough was observed at approximately 2 mg for CF4M (2.02) and CF3-4M (1.96). For CF2-4M, perchlorate was detected in all effluent volumes. The total capacity before complete perchlorate breakthrough for the columns was 11.40 mg for CF4M, 9.51 mg for CF2-4M, and 13.75 mg for CF3-4M. Binding of all applied target in the initial applications is important to allow quantification of target in the original sample based on that recovered in the eluant. As a result, CF2-4M is a poor candidate for application in this format. The greater capacity of CF3-4M should provide a wider range of applicability for that sorbent. When considered in light of the ion binding ratios described above, CF3-4M offers the combination of features desirable for capture of perchlorate from natural water. Perchlorate breakthrough was similarly evaluated for the commercial resin Purolite A530E (Supporting Information, Figure S6). The perchlorate binding capacity of the A530E resin is much higher than that of the sorbents developed here. The column breakthrough studies indicate significant target breakthrough from the initial application. In addition, repeated application of the 0.2 M HCl eluent resulted in recovery of only 27% of the bound target. 

**Figure 5:**
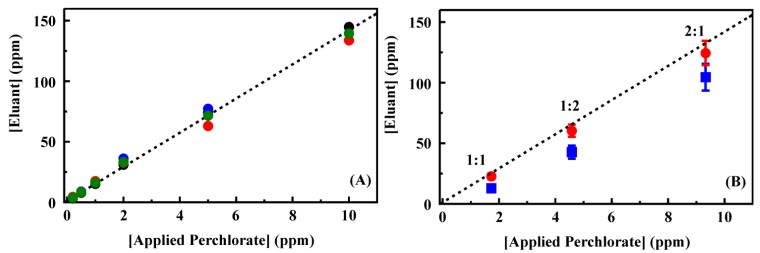

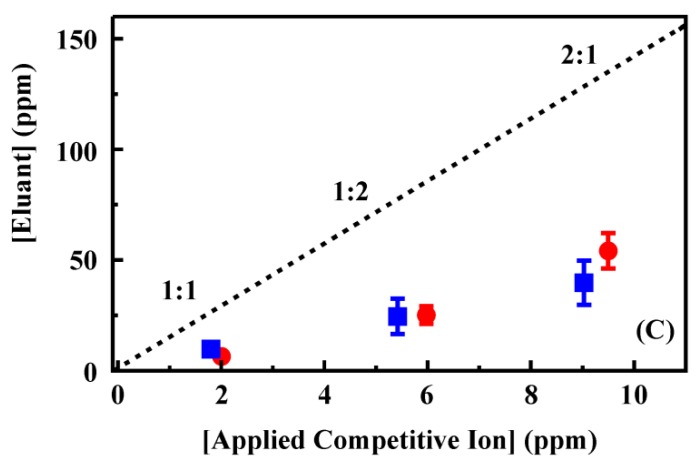
(**A**) Perchlorate recovery following preconcentration using a 200 mg column of CF3-4M. Target was applied to the column as a solution of the indicated concentration (50 mL). The column was then rinsed with deionized water (3 mL) before elution of the target in 0.2 M HCl (2 mL). Data points are the concentration of target recovered in the eluent volume for experiments completed in deionized water. The four data sets here were collected prior to (black, red) and following (blue, green) analysis of groundwater samples. A complete data set is provided in the Supporting Information, Table S4; (**B**,**C**) Recovery of perchlorate (**B**) and competitive ion (**C**) from mixed solutions in deionized water following preconcentration using a 200 mg column of CF3-4M. Data points are the concentration of ion recovered in the eluent volume for experiments completed using the indicate ratio of perchlorate to perrhenate (red circles) or thiocyanate (blue squares). Dashed lines indicate the fit from Figure 5A for comparison. Complete data sets are provided in the Supporting Information, Table S5.

A single CF3-4M column was used for preconcentration of perchlorate from a series of 100 samples including single and two target spiked deionized water and groundwater samples. No degradation in column performance was observed over the series. Degradation in column performance, reduced target retention, was noted beyond 130 repeated uses (data not shown). Targets were applied as 50 mL samples and elution was accomplished using 2 mL 0.2 M HCl. Figure 5 shows the eluant concentrations for perchlorate applied in deionized water. The complete data set is provided in the Supporting Information, Table S4. Complete binding of target and full recovery in the eluant volume would have resulted in a concentration enhancement of 25 times. Here, the concentration was enhanced by only 14 times (on average) following preconcentration. A linear dependence between concentration applied and that in the eluant was, however, observed for perchlorate. For these experiments, all perchlorate was bound from samples at less than 2 ppm. Some target was detected in effluent and first wash volumes for target concentrations at 5 and 10 ppm. For the higher target concentrations, perchlorate was also detected in the final HCl purge volume. This residual perchlorate indicates that an increased concentration of HCl may result in greater concentrations in the eluant volume. Unfortunately, the IC method applied here could not be applied with increased HCl concentrations as broadening of the chloride peak interfered with analysis of perchlorate concentrations. 

A series of samples containing mixtures of perchlorate and perrhenate or thiocyanate at varying concentrations was prepared (Figure 5) to evaluate the impact of target mixtures on column performance. Total ion concentrations of 4 and 15 ppm were used for which ratios of 1:1, 2:1 and 1:2 perchlorate to competing ion were prepared. Under these conditions, the recovery of perchlorate in the presence of perrhenate (Figure 5) was not statistically different from perchlorate recovery from single target solutions (within 1.5 standard deviations from the mean). The presence of thiocyanate, however, did significantly reduce the recovery of perchlorate. In the eluant volumes for these samples, both perrhenate and thiocyanate concentrations were enhanced by less than the perchlorate concentration enhancement (Figure 5, complete data set provided in the Supporting Information, Table S5). 

### 2.3. Groundwater Samples

When spiked groundwater samples were analyzed, little or no target was retained by the CF3-4M sorbent column. Analysis of perchlorate in other matrices (pond water, tap water, barrel collected rain water and soil extracts) showed similar results with little or no retained target. Column degradation was initially suspected, but additional analysis of samples in deionized water returned the expected results (Figure 5). Analysis of the water samples by IC indicated varying concentrations of sulfates, phosphates, and chloride, but not at levels that would be expected to fully prevent perchlorate binding. It was possible to achieve some perchlorate binding through dilution of the water samples (in deionized water) indicating the presence of other competing compounds. It was not clear whether this competition was the result of other small ionic targets not included in the IC analysis or to proteins and other organic compounds which may adsorb to the charged surface of the sorbents. The commercial sorbents retained perchlorate at levels similar to those observed for deionized water samples (Supporting Information, Table S8). 

**Figure 6:**
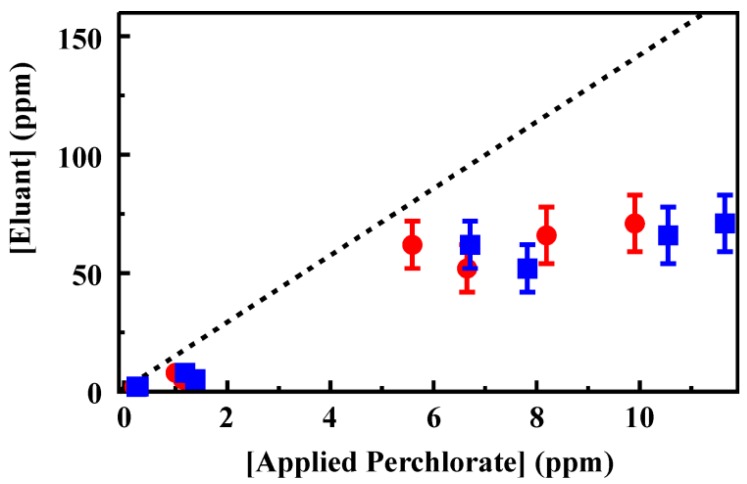
Perchlorate recovery from groundwater using a 200 mg column of CF3-4M. Target was applied to the column following preparation using a 200 mg activated charcoal column (50 mL). The column was then rinsed with deionized water (3 mL) before elution of the target in 0.2 M HCl (2 mL). Data points are the concentration of target recovered in the eluent volume *versus* the concentration recovered from the AC column (circles) and *versus* the initial target concentration (squares). Complete data sets are provided in the Supporting Information, Tables S6 and S7.

In order to address the difficulty presented by complex matrices, an activated charcoal (AC) preparatory step was utilized. The AC column was prepared identically to the CF3-4M column using 200 mg of the material. For the concentration range evaluated (0.2 to 100 ppm), 85% of the perchlorate passed through the AC column (Supporting Information, Table S6). When effluent recovered from the AC column was applied to the CF3-4M column, perchlorate was retained. Elution using HCl as described above resulted in enhanced perchlorate concentrations; however, the enhancements were not as significant as those observed for samples in deionized water (Figure 6; Supporting Information, Table S7). Sampling from the groundwater matrix resulted in increased noise in the baseline of IC chromatographs leading to an increased limit of detection for perchlorate and greater difficulty in analysis of low concentrations. As a result, concentrations in effluent, wash, and purge steps were not detected for several of the samples evaluated. It is likely that reduced enhancement is due to decreased target retention in these samples even following the AC step. As an alternative to the AC preparatory step, samples were treated with hydrogen peroxide [26]. For this process, spiked groundwater samples were prepared as above at 100 ppm and 50 ppm. The samples were then diluted 1:3 using either 30% hydrogen peroxide or deionized water. A larger percentage of target was retained from samples treated with peroxide (10%). This result further supports the likely interaction of organic carbon in the samples with the sorbent surfaces. 

## 3. Experimental Section 

*N*-trimethoxysilylpropyl-*N*,*N*,*N*-trimethylammonium chloride (TSPMC, 50% in methanol), and *N*-trimethoxysilylpropyl-*N*,*N*,*N*-tri-n-butylammonium chloride (TSPBC, 50% in methanol) were obtained from Gelest, Inc. Sodium perchlorate, sodium perrhenate, ammonium nitrate, ammonium thiocyanate, ammonium sulfate, ammonium phosphate, activated charcoal (catalog #05113), tetramethyl orthosilicate 98% (TMOS), hydrochloric acid 37% (HCl), nitric acid 70% (HNO_3_), and mesitylene(1,3,5-trimethylbenzene, TMB) were obtained from Sigma-Aldrich. Ethanol (200 proof) was obtained from the Warner-Graham Company. Pluronic^®^ P123 was donated generously by BASF (can be purchased from Sigma-Aldrich as poly(ethylene glycol)-*block*-poly(propylene glycol)-*block*-poly(ethylene glycol) average *M*_n_ ≈ 5800). Purolite^®^ A530E and A532E strong base anion exchange resins were gifts from Purolite (Bala Cynwyd, PA). Chemicals were used as received. Water was deionized to 18.2 MΩ cm using a Millipore Milli-Q UV-Plus water purification system. 

### 3.1. Material Synthesis 

The materials utilized in this effort were synthesized based on a previously published approach [18,19]. For synthesis of the HX sorbent, 4.0 g of Pluronic P123 and 0.85 g of TMB were dissolved in 12.0 g of 1.0 M HNO_3_ with magnetic stirring and heating (*ca*. 60 °C). The stirring mixture was allowed to cool to room temperature and 5.15 g of TMOS was added drop-wise. The mixture was stirred until homogeneous, transferred to a culture tube or Teflon jar, sealed tightly, and heated at 60 °C over night (≥18 h). The white monolith was dried in the unsealed tube at 60 °C for approximately 5 days. Surfactant was removed by calcination under ambient atmosphere, with temperature ramped 1 °C/min to 650 °C and held for 5 h. Other sorbents were prepared identically with the exception of the TMB used: 3.10 g for CF, 1.25 g for CF3, and 0 g for CF2. Materials were dried at 110 °C prior to grafting with alkylammonium silanes. Sorbent (1 g) was added to 100 mL of toluene followed by addition of a designated amount of TSPMC and/or TSPBC. The mixture was refluxed for 24 h. Grafted product was collected by vacuum filtration, washed with toluene then ethanol, and dried at 110 °C. The nomenclature of functionalized materials reflects the amounts of TSPMC and/or TSPBC used in the grafting procedure. For example, material designated HX1M3B was prepared by refluxing 1 g of HX with 1 mmol of TSPMC (M) and 3 mmol of TSPBC (B).

Nitrogen adsorption experiments were performed on a Micromeritics ASAP 2010 porosimeter at 77 K (Micromeritics Instrument Corporation, Norcross, GA). Samples were degassed to 1 µm Hg at 100 °C prior to analysis. Surface area was determined by use of the Brunauer-Emmett-Teller (BET) method, pore size was calculated by the Barrett-Joyner-Halenda (BJH) method from the adsorption branch of the isotherm, and total pore volume was determined by the single point method at relative pressure (*P*/*P*_0_) 0.97. Powder X-ray diffraction patterns were collected at room temperature using CuKα radiation from a Brüker MICROSTAR-H X-ray generator operated at 40 kV and 30 mA equipped with a 3 mRadian collimator, and a Brüker Platinum-135 CCD area detector. A custom fabricated beamstop was mounted on the detector to allow data collection to approximately 0.4° 2*θ* (approximately 210 Å) with a sample to detector distance of 30 cm. After unwarping the images the XRD^2^ plug-in was used to integrate the diffraction patterns from 0.6° to 8° 2*θ*. Scanning electron microscopy (SEM) was performed with a LEO 1455 SEM (Carl Zeiss SMT, Inc., Peabody, MA). A tungsten filament and secondary electron detector were used at a beam voltage of 15–20 kV. Samples were mounted on SEM stubs using conductive carbon tape and sputter coated with gold or palladium using a Cressington 108 auto sputter coater for 60 s. 

### 3.2. Target Binding and Elution

Column and batch type experiments were used to characterize the binding capacity and affinity of the sorbent materials. Batch experiments were conducted in 20 mL scintillation vials (EPA Level 3; clear borosilicate glass; PTFE/silicone-lined cap) using a fixed mass of sorbent (indicated in text and figure captions). Target samples (5 mL) were prepared in 18.2 MΩ Milli-Q deionized water or in groundwater as indicated. Target solutions were added to the sorbents in the vials with a portion of the sample retained for use as a control during IC analysis. Serial dilution of the retained sample was used for generation of a standard curve. Vials were incubated overnight (unless otherwise indicated) on rotisserie mixers. All samples were filtered using 25 mm Acrodisc 0.2 μm syringe filters with PTFE membranes prior to IC analysis. Difference method analysis was applied to determine the target removed from solution. Sorbent columns were prepared in Omnifit borosilicate column housings (6.6 mm × 50 mm, Diba Industries, Mahopak, NY, USA). Sorbent mass is provided in the text and figure captions. Controlled flow (1 mL/min) experiments were conducted. As with batch experiments, IC difference analysis was used to determine target bound.

### 3.3. Ion Chromatography 

Ion chromatography (IC) was used for analysis of perchlorate and other ionic targets. US Environmental Protection Agency method 314.0 was adapted for this purpose [17]. Analysis was carried out on a Dionex ICS 1000 system using suppressed conductivity. An anion self-regeneration suppressor (ASRS 300 4 mm) was used with 50 mM KOH as the eluent at 1 mL/min. For analysis of sulfate, the KOH eluent concentration was reduced to 25 mM. The stationary phase was an Ion Pac AS23 Analytical 4 mm × 250 mm column. An applied current of 200 mA was used with an injection volume of 250 μL and a cell temperature of 35 °C. Six point target calibration curves were used, and stock target concentrations were measured as a reference for each experiment. The limit of detection for the method was 0.1 ppm for samples in deionized water. For samples in complex matrices, the limit of detection was higher (2 ppm) due to increased baseline noise. 

## 4. Conclusions 

Binding of perchlorate and other ionic targets was found to vary significantly between silicate sorbents functionalized with combinations of two alkylammonium groups. Differences were observed even for sorbents functionalized at similar loading levels of identical groups. From these results, it seems that factors such as variations in diffusion, surface curvature, or site crowding may influence the performance of the sorbents. Through combinations of these effects it was possible to generate a sorbent with affinity for perchlorate that was higher than that for other ionic targets such as perrhenate. The selected sorbent was applied in a column format to extraction of perchlorate from spiked solutions including those with two targets and those prepared from groundwater samples. While perchlorate binding capacity in the sorbents developed here was less than that of the commercial resins, these sorbents offer several advantages. First, the silicate sorbents favor perchlorate binding over perrhenate; Purolite resins favor perrhenate. Second, the silicate sorbents more effectively capture perchlorate at low concentrations simplifying quantitative analysis of concentrations. Finally, elution of perchlorate from the silicate sorbents can be achieved using only hydrochloric acid. This eliminates the need for the post-processing steps necessary for iron containing eluents utilized with commercial resins. Unfortunately, other constituents in groundwater significantly reduced target retention in the sorbents. Passing samples through an activated charcoal column prior to analysis using the silicate column improved performance, but did not fully address the differences. 

EPA Method 314 is widely utilized for perchlorate analysis. This method is based on ion chromatography and provides a detection limit of approximately 0.2 μg/L, well below the standard of 2 ppb set by the Massachusetts Department of Environmental Protection. For laboratory analysis of this type, concentration of perchlorate and separation from other ions is not necessary. While offsite analysis of samples is the standard for evaluating sites of interest, this type of sample collection and analysis process is both expensive and time consuming. Onsite methods as indicators of the need for further testing and/or *in situ* methods for continuous monitoring of contamination levels are desirable. Ion selective electrodes provide an alternative to laboratory based analysis offering the potential for this portability. One such electrode from ELIT (8061) provides a limit of detection of 200 ppb with low levels of interference by other ions. The sorbents described here could be used to provide preconcentration of perchlorate ahead of this type of detection offering performance in lower concentration ranges in a portable format. This type of application would require a preparatory step, such as that provided by the activated charcoal column, to eliminate interfering components from the natural water samples. The sorbents may also offer utility in isotopic analysis and source identification as a result of preferential target binding [6,7]. 

## Supplementary Materials

Supplementary File 1
