# Supplementary material for: Extraction of Perchlorate Using Porous Organosilicate Materials
Source: Materials (Basel). 2013 Apr 2;6(4):1403–19. doi: 10.3390/ma6041403 (PMC5452307; doi:10.3390/ma6041403)
Supplement: Supplementary File 1 [file materials-06-01403-s001.pdf]

Supplementary Information

This supporting information includes additional morphological characterization and additional target adsorption data.

Figure S1. Nitrogen sorption isotherms and pore size distributions for grafted HX (**A**,**C**) and CF (**B**,**D**) sorbents. Panel A—HX05M05B (black) shifted by +450, HX 1M1B (red) shifted by +390, HX2M (blue) shifted by +200, HXB4 (green) shifted by +110, HX4M (purple), HX2M2B (orange) shifted by −90; Panel B—CF05M05B (black) shifted by +450, CF 1M1B (red) shifted by +300, CF2M (blue) shifted by +170, CFB4 (green), CF4M (purple), CF2M2B (orange) shifted by −160; Panel C—colored as in Panel A. Panel D—colored as in Panel B.

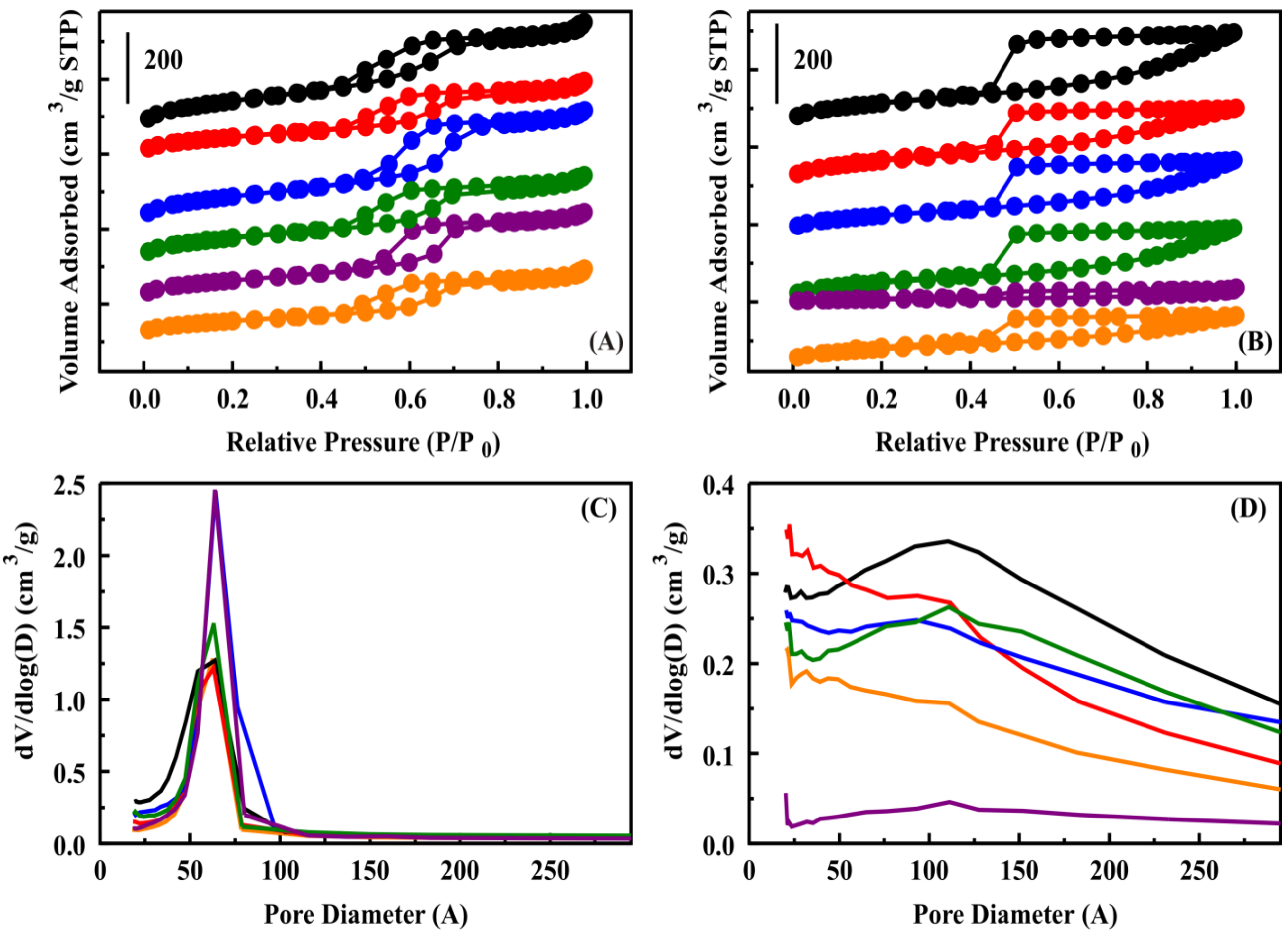

Figure S2. Nitrogen sorption isotherms (**A**) and pore size distributions (**B**) for CF2 (green) and CF3 (black, shifted +390) and the grafted variants CF2-4M (red) and CF3-4M (blue, shifted +370).

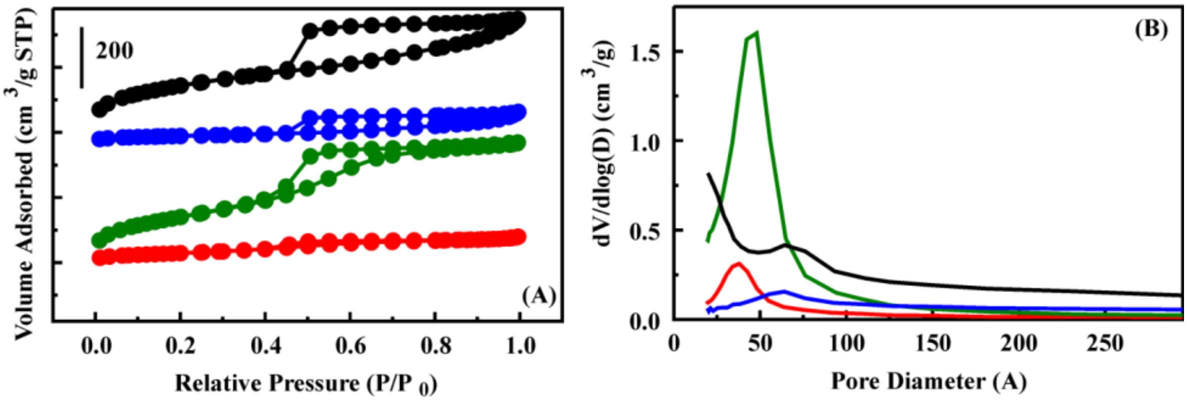

Figure S3. Scanning electron microscope (SEM) images of CF2 (**A**) and CF3 (**B**) showing differing macroscale morphologies.

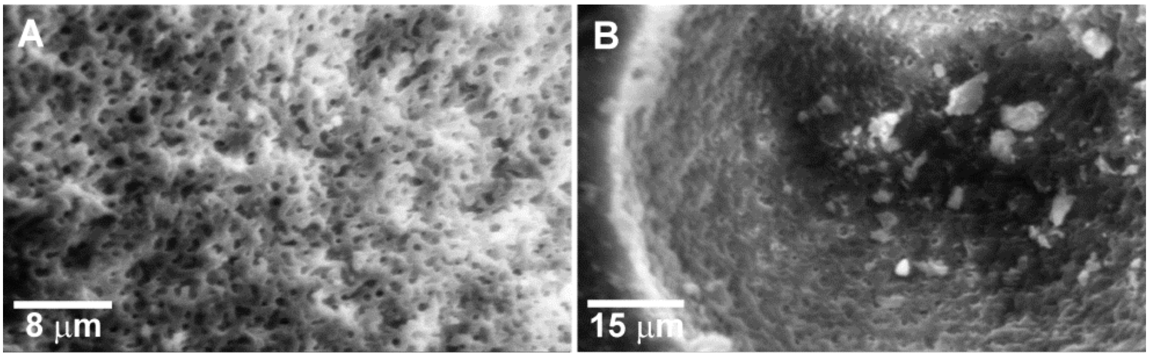

Table S1. Summary of ions bound by materials variants using 10 mg of sorbent in a 200 ppm solution (5 mL).

Material	Target Bound (µg)					
	**Perchlorate**	**Perrhenate**	**Nitrate**	**Thiocyanate**	**Sulfate**	**Phosphate**
**HX Products**						
HX2M	239	333	114	166	311	200
HX1M3B	216	547	116	84	146	178
HX2M4B	54	330	68	20	121	164
**CF Products**						
CF4M	188	359	101	112	191	199
CF1M1B	339	633	158	180	78	168
CF2-4M	168	137	60	134	197	163
CF3-4M	258	257	91	191	250	191
**Purolite Products**						
A530E	670	1000 *	610	750	–	270
A532E	680	1000 *	760	750	–	270

* 100% of target bound; when concentrations of targets were increased to 500 ppm, the Purolite resins also bound 100% of perrhenate (2510 µg), but only 1740 µg (A530E) and 650 µg (A532E) perchlorate.

Table S2. Ratio of perchlorate bound to ions bound by materials variants using 10 mg of sorbent in a 200 ppm solution (5 mL).

Material	Target Ratio				
	ClO_4/ReO_4	ClO_4/NO_3	ClO_4/SCN	ClO_4/SO_4	ClO_4/PO_4
			**HX Products**		
HX2M	0.72	2.10	1.43	0.77	1.20
HX1M3B	0.39	1.86	2.57	1.48	1.21
HX2M4B	0.16	0.79	2.70	0.45	0.33
			**CF Products**		
CF4M	0.52	1.86	1.68	0.98	0.94
CF1M1B	0.54	2.14	1.88	4.35	2.02
CF2-4M	1.23	2.80	1.25	0.85	1.03
CF3-4M	1.00	2.84	1.35	1.03	1.35
			**Purolite Products**		
A530E	0.67*	1.10	0.89	–	2.48
A532E	0.68*	0.89	0.91	–	2.52

* Artificially limited by experimental conditions. When target concentrations were increased to 500 ppm for perchlorate and perrhenate, target bound for A530E was increased to 1.74 and 2.39 mg, respectively indicating a ratio of 0.73. For A532E under these conditions, binding of perchlorate was 0.68 mg and perrhenate was 2.37 mg giving a ratio of 0.28.

Table S3. Radii for ionic targets considered in these studies.

Ion	Radius (Å)				References
	Crystal	Stokes	CSD [1]	Ionic [2]	
Perchlorate	–	1.37	1.40(6)	1.81	[3]
Perrhenate	2.60	1.79	1.70(3) *	0.56	[4]
Phosphate	–	–	1.50(4)	0.38	–
Sulfate	–	–	1.47(3)	0.37	–
Nitrate	1.79	1.40	1.24(6)	0.13	[4]
Thiocyanate	2.13	1.58	1.39(5) §	–	–

* search screens refined to select for ionic form only; § reported value is half the S–N distance.

Figure S4. Perchlorate binding from batch experiments by HX4M (**A**), CF2-4M (**B**), CF3-4M (**C**), CF4B (**D**), CF2M2B (**E**), CF1M1B(**F**), HX1M1B (**G**), CF2M (**H**), HX2M (**I**), HX2M2B (**J**), CF1M3B (**K**), HX1M3B (**L**). Shown are experimental data (black circles) as well as the results of fitting that data (red ×).

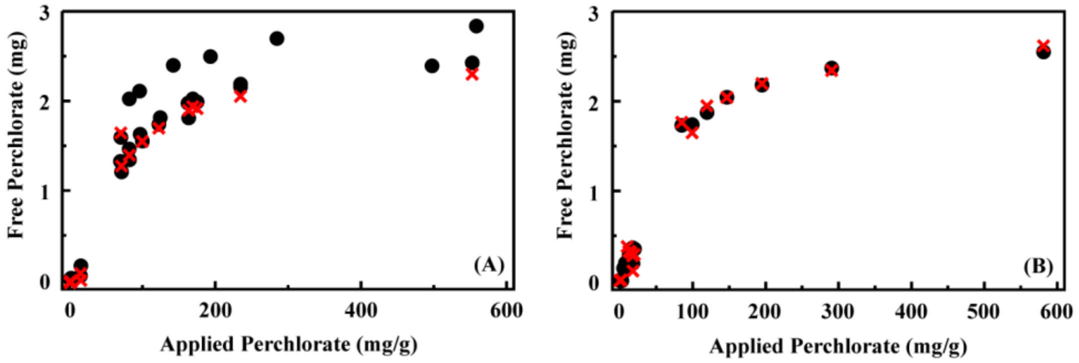

Figure S4. *Cont.*

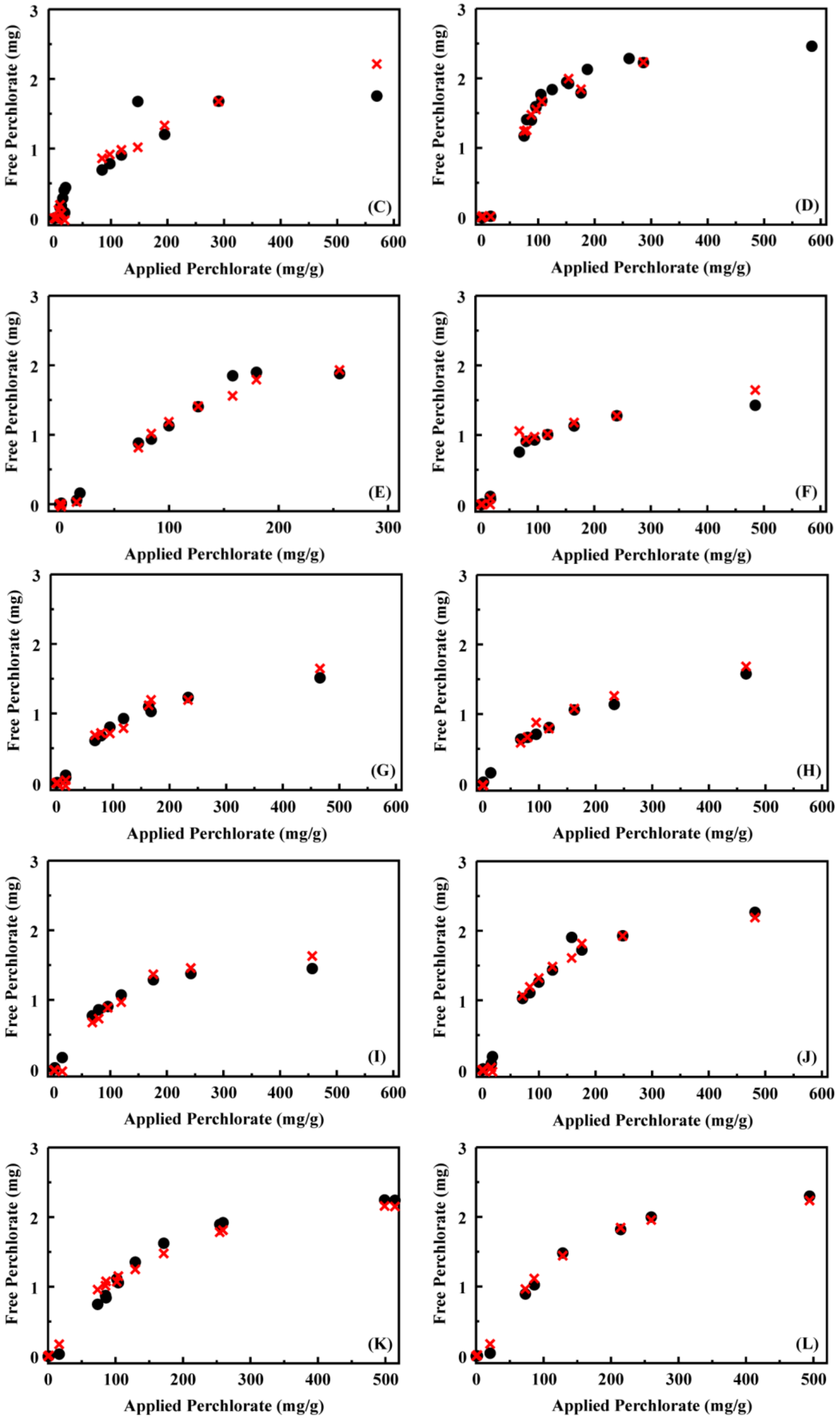

Figure S5. Competitive ion binding from mixed target solutions. Data for each axis is plotted as the ratio of the target bound from the two target solution to that bound from the single target solution: binding from solutions of perchlorate and perrhenate (red circles) and binding from solutions of perchlorate and thiocyanate (black squares). Target ratios of 4:1, 1:1, and 1:4 were utilized. (**A**) CF4M; (**B**) CF2-4M; (**C**) CF1M3B; (**D**) HX1M3B; (**E**) HX2M.

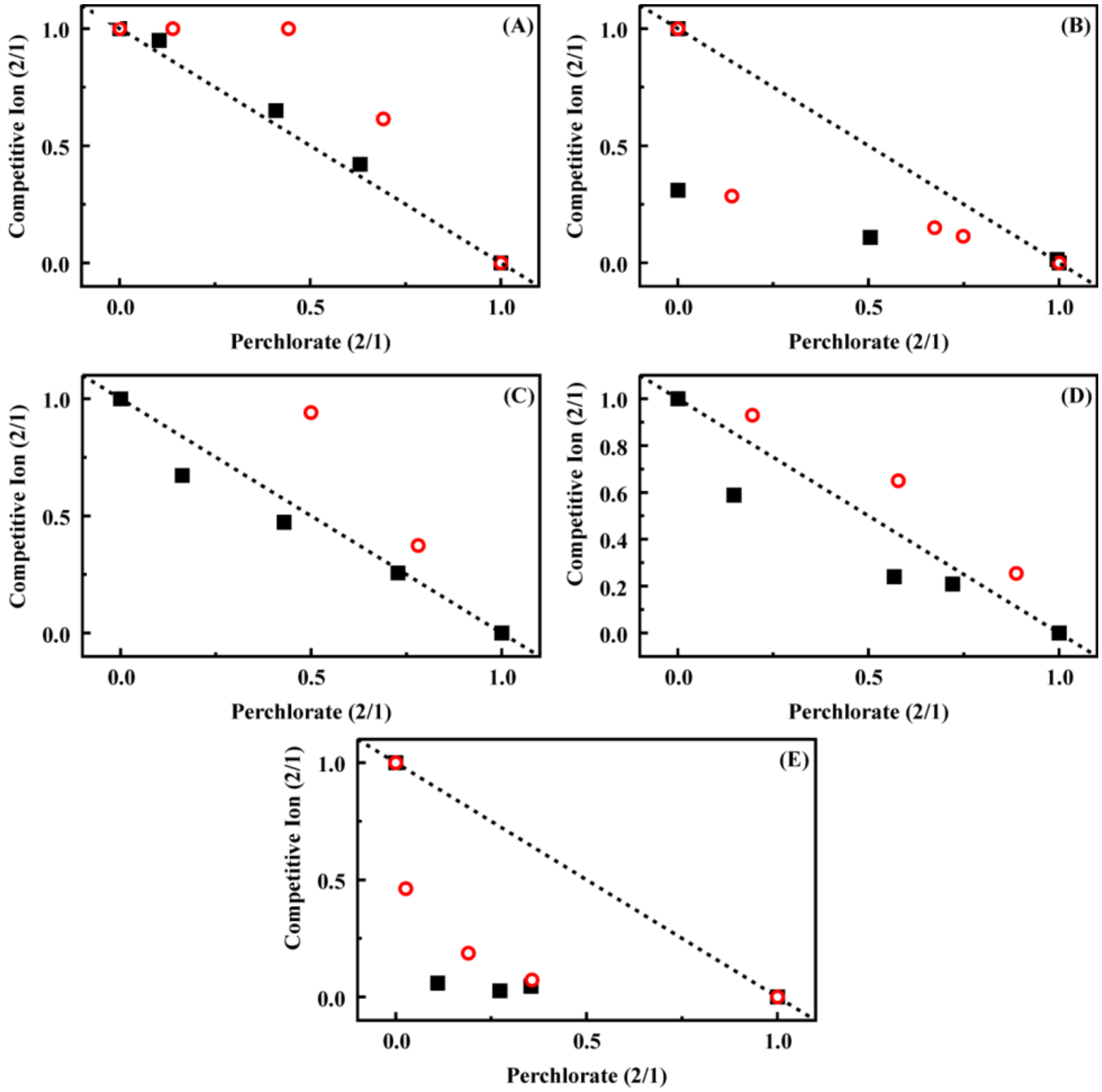

Figure S6. (**A**) Perchlorate breakthrough for 50 mg column of Purolite A530E using a 10 ppm solution at a flow rate of 1 mL/min; (**B**) Chloride recovered in volumes collected during breakthrough experiment. Dashed lines are from data for CF4M (Figure 4). The capacity of the commercial sorbent is much greater than that of the CF sorbents developed for this study. The data for this column, however, shows significant target breakthrough from the initial application. Total target bound across these additions was 3.74 mg; (**C**) Repeated applications of 0.2 M HCl (5 mL each for a total of 35 mL) recovered only 27% of the total target bound.

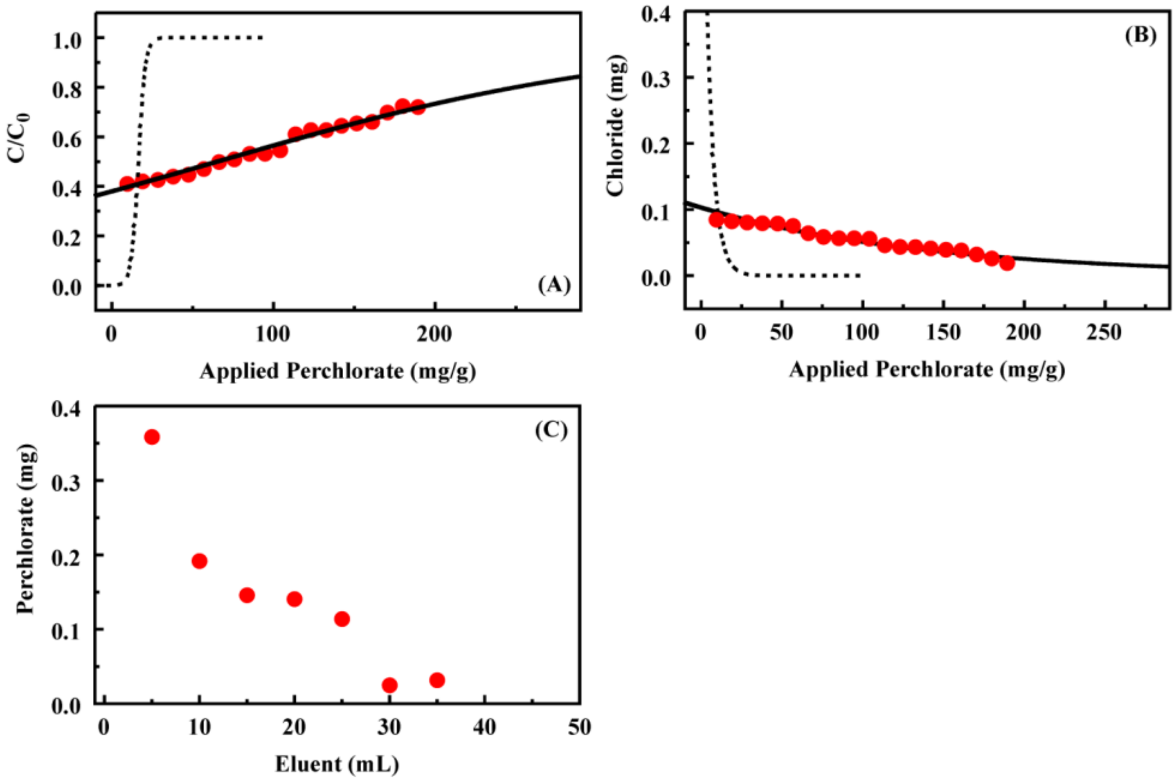

Table S4. Perchlorate preconcentration from deionized water. The target recovered in each volume for perchlorate application and recovery from a column of CF3-4M. Target was applied to the column as a solution in 50 mL of deionized water at 1 mL/min (effluent). The column was then rinsed with 3 mL of deionized water (rinse 1) before elution of the target in 2 mL of 0.2 M HCl (eluent). The column was purged with 10 mL of 0.2 M HCl (purge) and rinsed with 3 mL deionized water (rinse 2) before application of the next target sample.

Target	unit	Applied	Effluent	Rinse 1	Eluent	Purge	Rinse 2	Total
0.2 ppm	μg	10.7	0	0.14	6.60	2.81	0	9.55
	ppm	0.2	0	0.05	**3.30**	0.28	0	–
	%	–	0	1	62	26	0	89
0.5 ppm	μg	26	2.14	0.25	15.85	8.03	0	26.26
	ppm	0.5	0.04	0.08	**7.92**	0.80	0	–
	%	–	8	1	62	31	0	102

Table S4. *Cont.*

1 ppm	μg	50	4.70	0.51	30.46	11.96	0	47.63
	ppm	1.0	0.09	0.17	**15.23**	1.20	0	–
	%	–	9	1	61	24	0	95
2 ppm	μg	99	13.43	1.08	62.08	24.13	0	100.72
	ppm	2.0	0.27	0.36	**31.04**	2.41	0	–
	%	–	14	1	63	24	0	102
5 ppm	μg	250	52.95	3.66	148.59	46.35	0	252
	ppm	5.0	1.06	1.22	**74.30**	4.64	0	–
	%	–	21	1	59	19	0	101
10 ppm	μg	500	115.30	0.39	289.62	9.61	0	501
	ppm	10	2.31	0.13	**144.81**	96.08	0	–
	%	–	23	0	58	19	0	100

Table S5. Perchlorate preconcentration from mixed target solutions. The target recovered in each volume for perchlorate plus competing ion application and recovery from a column of CF3-4M. Target was applied to the column as a solution in 50 mL of water at 1 mL/min (effluent). The column was then rinsed with 3 mL of deionized water (rinse 1) before elution of the target in 2 mL of 0.2 M HCl (eluent). The column was purged with 10 mL of 0.2 M HCl (purge) and rinsed with 3 mL deionized water (rinse 2) before application of the next target sample.

Target mixture	unit	Applied	Effluent	Rinse 1	Eluent	Purge	Rinse 2	Total
2 ppm perchlorate + 2 ppm perrhenate	μg	84.64	33.05	1.89	38.74	5.17	0	78.86
	ppm	1.69	0.66	0.63	19.37	0.52	0	–
	%	–	39	2	46	6	0	93
	μg	85.54	89.81	0	21.84	0	0	61.64
	ppm	1.71	0.80	0	10.92	0	0	–
	%	–	47	0	26	0	0	72
10 ppm perchlorate + 5 ppm perrhenate	μg	462.34	207.02	14.81	239.35	15.42	0	476.59
	ppm	9.25	4.14	4.94	119.67	1.54	0	–
	%	–	45	3	52	3	0	103
	μg	284.73	107.72	7.43	97.52	4.60	1.68	218.64
	ppm	5.69	2.15	2.48	48.76	0.46	0.46	–
	%	–	38	3	34	2	0	77
5 ppm perchlorate + 10 ppm perrhenate	μg	238.38	9.68	6.46	115.76	6.87	0	228.77
	ppm	4.77	1.99	2.15	57.88	0.69	0	–
	%	–	42	3	49	3	0	95.97
	μg	508.72	293.62	19.31	205.03	6.56	1.38	525.88
	ppm	10.17	5.87	6.44	102.51	0.65	0.46	–
	%	–	58	4	40	1	0	103
2 ppm perchlorate + 2 ppm thiocyanate	μg	84.57	33.88	2.02	38.93	0	0	74.83
	ppm	1.69	0.68	0.67	19.47	0	0	–
	%	–	40	2	46	0	0	88
	μg	91.70	43.42	3.30	37.64	5.57	0	89.93
	ppm	1.83	0.87	1.10	18.82	0.56	0	–
	%	–	47	4	41	6	0	98
10 ppm perchlorate + 5 ppm thiocyanate	μg	426.25	214.34	10.66	222.18	10.60	0	457.78
	ppm	8.53	4.26	3.55	111.09	1.06	0	–
	%	–	50	3	52	2	0	107
	μg	218.90	119.92	5.74	91.31	8.89	0	225.86
	ppm	4.38	2.40	1.91	46.65	0.89	0	–
	%	–	55	3	42	4	0	103

Table S5. *Cont.*

5 ppm perchlorate + 10 ppm thiocyanate	µg	246.12	113.86	21.62	79.05	0	0	214.53
	ppm	4.92	2.28	7.21	39.53	0	0	–
	%	–	46	9	32	0	0	87
	µg	474.76	234.37	45.60	149.43	9.63	0	439.02
	ppm	9.50	4.69	15.20	74.71	0.96	0	–
	%	–	49	10	31	2	0	92

Table S6. Perchlorate recovered from activated charcoal preparatory step in parts per million (ppm).

Applied	Effluent	Variation
0.28	0.23	0.02
1.38	1.14	0.03
6.71	5.59	0.15
10.55	8.19	1.01
46.30	40.53	1.03
88.30	81.49	4.71

Table S7. Perchlorate preconcentration from groundwater. The target in each volume for perchlorate application and recovery from a column of CF3-4M. Target was applied to the column as a 50 mL solution recovered following the AC preparatory column (Table 6; 1 mL/min; effluent). The column was then rinsed with 3 mL of deionized water (rinse 1) before elution of the target in 2 mL of 0.2 M HCl (eluent). The column was purged with 10 mL of 0.2 M HCl (purge) and rinsed with 3 mL deionized water (rinse 2) before application of the next target sample.

Target	unit	Applied	Effluent	Rinse 1	Eluent	Purge	Rinse 2	Total
0.2 ppm	µg	11.5	0	0	4.1	0	0	4.1
	ppm	0.2	0	0	**2.1**	0	0	–
	%	–	0	0	36	0	0	36
1 ppm	µg	57	0	0	10	0	0	10
	ppm	1.1	0	0	**5.2**	0	0	–
	%	–	0	0	18	0	0	18
5 ppm	µg	333	0	0	104	0	0	104
	ppm	6.65	0	0	**52**	0	0	–
	%	–	0	0	31	0	0	31
10 ppm	µg	411	96	0	132	0	0	228
	ppm	8.2	1.9	0	**66**	0	0	–
	%	–	23	0	32	0	0	55

Table S8. Perchlorate preconcentration from groundwater using Purolite resins. The target recovered in each volume for perchlorate application and recovery. Target was applied to the column as a solution in 50 mL of water at 1 mL/min (effluent). The column was then rinsed with 3 mL of deionized water (rinse 1) before elution of the target in 20 mL of 0.2 M HCl (eluent). The column was rinsed with 5 mL deionized water (rinse 2) before application of the next target sample. Groundwater was collected from a household wells in Fulton, MD, USA (depth of 213 m) and filtered using a 0.7 µm filter flask before spiking with the indicated target concentration.

Material	Target	Unit	Applied	Effluent	Rinse 1	Eluent	Rinse 2	Total
Purolite A530E	10 ppm	µg	500	34.12	0	90.71	0.53	125.35
		ppm	10	0.68	0	4.53	0.11	–
		%	–	7	0	18	0	26
Purolite A530E	50 ppm	µg	2500	108.01	0.16	105.06	0.71	213.94
		ppm	50	2.16	0.05	5.25	0.14	–
		%	–	5	0	4	0	9
Purolite A532E	10 ppm	µg	500	10.25	0	8.48	0	18.73
		ppm	10	0.21	0	0.42	0	–
		%	–	2	0	2	0	4
Purolite A532E	50 ppm	µg	2500	44.04	0	10.01	0	54.05
		ppm	50	0.88	0	0.50	0	–
		%	–	2	0	0	0	2

References

1. Orpen, A.G. Applications of the Cambridge Structural Database to molecular inorganic chemistry. *Acta Crystallogr. B* **2002**, *58*, 398–406.
2. Barbalace, K. Periodic table of elements—Sorted by ionic radius. Available online: http://EnvironmentalChemistry.com/yogi/periodic/ionicradius.html (accessed on 1 April 2013)
3. Yoon, J.; Yoon, Y.; Amy, G.; Cho, J.; Foss, D.; Kim, T.-H. Use of surfactant modified ultrafiltraion for perchlorate ClO_4^- removal. *Water Res.* **2003**, *37*, 2001–2012.
4. Mbuna, J.; Takayanagi, T.; Oshima, M.; Motomizu, S. Eavluation of weak ion association between tetraalkylammonium ions and inorganic ions in aqueous solutions by capillary zone electrophoresis. *J. Chrom. A.* **2004**, *1022*, 191–200.

© 2013 by the authors; licensee MDPI, Basel, Switzerland. This article is an open access article distributed under the terms and conditions of the Creative Commons Attribution license (http://creativecommons.org/licenses/by/3.0/).
